# A double blinded placebo controlled comparative clinical trial to evaluate the effectiveness of Siddha medicines, Kaba Sura Kudineer (KSK) & Nilavembu Kudineer (NVK) along with standard Allopathy treatment in the management of symptomatic COVID 19 patients - a structured summary of a study protocol for a randomized controlled trial

**DOI:** 10.1186/s13063-021-05041-x

**Published:** 2021-02-11

**Authors:** Anurag Srivastava, Manickavasagam Rengaraju, Saurabh Srivastava, Vimal Narayan, Vivek Gupta, Rashmi Upadhayay

**Affiliations:** 1 Government Institute of Medical sciences (GIMS), Greater Noida, UP 201310 India; 2 Siddha Clinical Research Unit, Safdarjung Hospital, A Unit of Central Council for Research in Siddha, New Delhi, 110029 India

**Keywords:** COVID 19, Randomised Controlled Trial, Protocol, Siddha Medicine, Herbal, CAM

## Abstract

**Objectives:**

The primary objectives of the study are to determine the effectiveness of the Kaba Sura Kudineer (KSK) & Nilavembu Kudineer (NVK) along with standard Allopathy Treatment to compared with Placebo (Decaffeinated Tea) with standard Allopathy Treatment in the management of Symptomatic COVID 19 patients and also in reduction of Hospital Stay Time & Changes in Immunological (IL6) and Bio Chemical Markers (Ferritin, CRP, D-Dimer and LDH).

The secondary objectives are to evaluate the safety of the trial medicines and their effects in the reduce the risks of the disease. In addition, to document the profile of Symptomatic COVID 19 patients as per Siddha Principles.

**Trial Design:**

A Double Blinded, Three arm, Single Centre, Placebo Controlled, Exploratory and comparative Randomized Controlled Trial

**Participants:**

Patients who were admitted to the COVID Care Centre at Govt. Institute of Medical Sciences. Noida in India will be recruited. These will be patients with Mild and Moderate symptoms with laboratory confirmed COVID 19 (RT – PCR Tested Positive) aged 18-65, willing and consenting to participate.

**Intervention and comparator:**

**Arm I:** Decaffeinated Tea (Placebo – similar in taste and appearance to the other Two Decoctions), 60 Ml Morning and Night after Food, along with standard Allopathy Treatment for 10 days.

**Arm II:** Nilavembu Kudineer (The Siddha Medicines which is used as a standard Anti-Viral drug for the past Pandemics by Siddha Physicians) 60 Ml Morning and Night after Food, along with standard Allopathy Treatment for 10 days.

**Arm III:** Kaba Sura Kudineer (The Siddha Medicine which is proposed to be used as a Treatment for COVID 19 based on Siddha Literature) 60 Ml Morning and Night after Food, along with standard Allopathy Treatment for 10 days.

The investigational drugs are registered products under the Govt.of India and bought from GMP Certified Manufacturing Units.

**Main Outcomes:**

**Primary outcomes:**

1. Reduction in Viral load of SARS-CoV-2 at the end of treatment (10 days). 2. Time taken to convert Patient from symptomatic to Asymptomatic based on Reduction in clinical symptoms (10 days). 3. Effect of drugs inflammatory markers (IL6,) at the end of treatment (10 days). 4. Reduction in hospital stay time (20 days follow up). (Based on RT PCR CT Value 3^rd^, 6^th^ if needed 10^th^ day). (Based on IL 6 Value needed 10^th^ day or IL6 value on turning negative. (entry level/exit level).

**Secondary outcomes (10 days):**

1. Reduction in use of Intensive Supportive Care. 2. Reduction in incidence of complications (Acute Respiratory Distress Syndrome, other systemic complications). 3. MuLBSTA score for viral pneumonia (multinodular infiltration, hypo-lymphocytosis, bacterial co infection, Total Leucocyte Count (TLC ≤ 0.8 x 10^9^/L), smoking history, hyper-tension and age) score. 4. Laboratory markers (Haematological & Biochemical Markers). 5. Adverse events/effects Siddha-based measurements. 6. Siddha Udaliyal assessment by using Yakkai Ilakkanam (YI) Tool to diagnose body condition for covid-19 patients.

**Randomisation:**

The assignment of the participants into 3 Groups will be allocated in 1:1:1 Ratio through randomization Blocks in Microsoft Excel by a Statistician who is not involved in the study. The allocation scheme will be made by another statistician by using a closed envelope after the assessment of eligibility and Informed consent procedures. The groups will be balanced for age and sex with 3:1 Ratio in each group for mild: severe COVID-19 symptoms.

**Blinding:**

The Study is Double Blinded. Participants and Investigators were blinded.

**Numbers to be randomized (Sample size):**

Sample size could not be calculated, Since there are no prior trials on KSK and NVK as a comparative trial. In addition, there are no prior trials on KSK and NVK in this region.

A total Number of 120 Patients, 40 each in 3 groups will be recruited in 1:1:1 Ratio.

**Trial Status:**

Protocol Number : SCRUND GIMS Noida Study 1,Version: 2.0 Protocol Date : 20.08.2020

The recruitment period is completed for the trial. The Trial started its recruitment on 22.8.2020. We anticipate study including data analysis will finish in January 2021.

This is to state that it was a late submission from authors for publication of the protocol to the BMC, after enrolment in the study was over.

**Trial Registration:**

The trial protocol was registered with CTRI (Clinical Trial Registry of India) and number is CTRI/2020/08/027286 on 21.08.2020

**Full Protocol:**

The full Protocol is attached as an additional file, Accessible from the Trials website (Additional file 1). In the interest in expediting dissemination of this material, the familiar formatting has been eliminated. This letter serves as a summary of the key elements of the full protocol. The Study protocol has been reported in accordance with the SPIRIT guidelines.

© The Author(s). 2021 **Open Access** This article is licensed under a Creative Commons Attribution 4.0 International License, which permits use, sharing, adaptation, distribution and reproduction in any medium or format, as long as you give appropriate credit to the original author(s) and the source, provide a link to the Creative Commons licence, and indicate if changes were made. The images or other third party material in this article are included in the article's Creative Commons licence, unless indicated otherwise in a credit line to the material. If material is not included in the article's Creative Commons licence and your intended use is not permitted by statutory regulation or exceeds the permitted use, you will need to obtain permission directly from the copyright holder. To view a copy of this licence, visit http://creativecommons.org/licenses/by/4.0/. The Creative Commons Public Domain Dedication waiver (http://creativecommons.org/publicdomain/zero/1.0/) applies to the data made available in this article, unless otherwise stated in a credit line to the data.

**Acknowledgements**
1. **Brigadier Dr. RakeshKumar Gupta,** Director, Government Institute of Medical sciences, (GIMS) Greater Noida, UP.
2. **Prof.Dr. K. Kanakavalli,** Director General, Central Council for Research in Siddha, Chennai.
3. **Prof.Dr. Jugal Kishore, Director,** HOD- Department of Community Medicine, VMMCH & Safdarjung Hospital, New Delhi.
4. **Dr.P. Sathyarajeshwaran,** Director- in-Charge, Siddha Central Research Institute, Central Council for Research in Siddha, Chennai.
RG,KK,JK and PS given their inputs to finalize the Study Protocol.

**Authors' Contributions**
MR, AS conceived the study. MR, AS and VN initiated the study. VG Contributed to incorporate all lab investigations. MR, AS, VN, SS and RV contributed to Protocol writing. This protocol was read and approved by all authors.

**Authors' Information**
AS,SS, RU and VG possess the background of Allopathy
MR and VN possess the background of Siddha (An Ancient Traditional medical system of India).

**Funding**
The Trial is funded by the Central Council for Research in Siddha (CCRS), Min.of AYUSH, Govt.of India and Government Institute of Medical sciences, (GIMS) Greater Noida, UP. The funding body had no role in the design of the study and collection, analysis and interpretation of data and in writing the manuscript.

**Ethics Approval and consent to participate**
The trial received the ethical approval from the Institutional Ethical Committee of Siddha Clinical Research Unit, Safdarjung Hospital, New Delhi on 20.07.2020 and Trial Site Ethics Committee on 04.08.2020.
This is to state that the appropriate ethical committee approval was taken. Written Consent will be taken from all eligible and willing participants before their participation.

**Consent for Publication**
Not Applicable

**Competing Interest**
The authors declare that they have no competing interests.

## Publisher's Note
Springer Nature remains neutral with regard to jurisdictional claims in published maps and institutional affiliations.

**Ready to submit your research? Choose BMC and benefit from:**

- fast, convenient online submission
- thorough peer review by experienced researchers in your field
- rapid publication on acceptance
- support for research data, including large and complex data types
- gold Open Access which fosters wider collaboration and increased citations
- maximum visibility for your research: over 100M website views per year

**At BMC, research is always in progress.**

**Learn more** biomedcentral.com/submissions

## Data Availability

All participant data will be kept confidential and personal identifiers of the study participants will be disclosed to the public. Only the Investigator will have access to the trial data. All the procedures will be carried out by adhering the Good Clinical Practices (GCP). The monitor will have access to the study documents.

